# The importance of disability representation to address implicit bias in the workplace

**DOI:** 10.3389/fresc.2023.1048432

**Published:** 2023-03-22

**Authors:** Daniel W. Derbyshire, Anne E. Spencer, Brit Grosskopf, Theo Blackmore

**Affiliations:** ^1^ European Centre for Environment and Human Health, University of Exeter, Exeter, United Kingdom; ^2^ Health and Community Sciences, University of Exeter Medical School, University of Exeter, Exeter, United Kingdom; ^3^ Department of Economics, University of Exeter, Exeter, United Kingdom; ^4^ disAbility Cornwall and Isles of Scilly, Truro, United Kingdom

**Keywords:** disability, implicit biases, human resources, workplace, employment

## Abstract

**Introduction:**

People are disabled by barriers in society, not by their impairment. Barriers can be physical or be caused by people's implicit and explicit attitudes towards people with disabilities.

**Methods:**

We utilise the Implicit Association Test to investigate implicit attitudes towards people with disabilities among Human Resource professionals and people involved in making hiring decisions.

**Results:**

We find no significant differences between people who work for large companies or Small- to Medium-sized Enterprises. Similarly, working in Human Resources (or making recruitment decisions) has no effect on implicit bias. We supply the first evidence linking a person's own health status (measured using EQ-5D-5L) to their implicit bias. We find that a worse health status is associated with lower implicit bias towards people with disabilities. In addition, we find women have lower implicit bias than men.

**Discussion:**

The discussion reflects on the need for greater disability representation within the workplace - especially in making hiring decisions.

## Introduction

While the employment gap of people with and without disabilities narrowed over recent years in the UK, there remains a persistent inequality. The UK employment rate for people with disabilities in the first quarter of 2022 was 53.8%, compared to 82.0% for people who do not have a disability. This equates to a disability employment gap of 28.2% (1). Within this context, disability refers to the Government Statistical Service definition that is used to determine disability status under the Equality Act (UK)—referring to physical or mental health conditions that are expected to last for 12 months or more and which reduce the ability to carry-out day-to-day activities (2). While this gap has narrowed over recent years from 33.8% in the first quarter of 2014 it remains persistent and significant [see (3)] despite substantial government interventions—from anti-discrimination legislation to active labour market policies. The UK has a labour force shortage of 1.3 million vacancies as of May 2022 (4). Given that there are over 8 million 16–64 years old people with disabilities in the UK [21.8% of the 16–64 population (1);], understanding the cause of the disability employment gap and creating workplaces that are more inclusive of people with disabilities could go some way towards bridging this labour shortage. Further highlighting the problems of barriers to employment facing people with disabilities, the unemployment rate for people with disabilities (6.6%) is more than twice the rate for those without a disability [3.2%, (1)].

The disparity in unemployment rates—the proportion of people wanting but unable to find work—highlights in particular the barriers that people with disability face in finding meaningful and continued employment. Through the lens of the social model of disability, the employment gap and unemployment disparity arise through societally imposed barriers that disable the work prospects of people with impairments.

In this paper, we focus on unconscious (or implicit) bias towards people with disabilities and as such, our study is situated at the nexus of taste-based discrimination models and the social model of disability. In particular, we provide the first evidence on the relationship between size of firm worked for and unconscious bias towards people with disabilities. Further, we present the first results investigating the effect that a person's own health status has on their attitudes towards people with disabilities.

## Framework and hypotheses

### The social model of disability

In the 40 years since it was first introduced, the social model of disability has revolutionised modes of thinking with respect to disability within academic fields, especially disability studies and rehabilitation sciences. The social model of disability is based on the premise that people with disabilities are not disabled by their own impairments but by inadequacies in the way society is set up to accommodate those with a disability (5). Within the context of the social model, people with a disability face barriers in equitable access to employment (6), education (7) and healthcare (8). The social model of disability contrasts with the medical model of disability, which focuses on the particular impairments of people with disabilities. There is increasing commitment to adhere to the social model of disability by policy makers [e.g., the National Disability Strategy (UK) explicitly mentions that the social model of disability is the underlying approach]. Further, Bunbury (9) posits that disability anti-discrimination legislation was largely written using the medical model of disability and that the social model of disability is necessary to properly understand and address these socially imposed barriers. Indeed, multiple studies have shown that the Disability Discrimination Act (UK) had no effect on the employment prospects of people with disabilities (10–12). The social model instead emphasises barriers are more structural and societal and therefore that simple anti-discrimination legislation is insufficient to address the problem (9). For example, Vedeler (13) presents evidence that job interviews/application process often involve procedures that are discriminatory towards people with disabilities. Beyond poorly designed applications processes and procedures, there are also attitudinal barriers that result in taste-based discrimination (14, 15). Taste-based discrimination refers to a situation in which discrimination is the result of a prejudice that affects a person's preferences for interacting with certain groups of people (e.g., people have a preference for people more similar to themselves) (16).

Studies that use taste-based discrimination approaches to examine the attitudinal barriers people with disabilities face in society include taste-based discrimination towards access to finance (e.g., people with disabilities face higher interest rates, lower credit limits and difficulty accessing life insurance) (17–19). Similarly, people with disabilities face barriers accessing accommodation (20, 21). Further, Villiger (22) highlights the importance of implicit attitudes in taste-based discrimination. Stigma and stereotyping are cited as a key barrier to employment for people with disabilities with persistent myths about lower productivity, high physical adaptation costs and high absenteeism [see (6) for an extensive review]. These incorrect assumptions can generate implicit preferences and taste-based discrimination. There are many key decision points in the process of obtaining and retaining employment that involve choosing among potential candidates, where there is room for implicit biases to enter the hiring processes. These include, but are not limited to, choosing who to interview, who to hire, who to promote, and, on occasion, who to fire. This is especially likely to be the case in small- to medium-sized enterprises (SMEs) who don't have dedicated Human Resource (HR) departments or budgets for engaging in equality, diversity and inclusion (EDI) activities, though we are not aware of any evidence relating to unconscious bias specifically in SME workplaces. This is a notable absence, since SMEs account for over 99% of businesses and over 50% of UK employment (23). Crucially, there is evidence that attitudes towards people with disabilities influence employers' willingness to comply with the Disability Discrimination Act (UK) legislation—which was later subsumed into the Equality Act (UK) (24).

### Implicit and explicit bias

According to the dual process model, cognitive processes occur simultaneously on implicit (or automatic) and explicit levels (25). It is also frequently observed that there is gap between self-reported (or explicit) attitudes and implicit attitudes as measured by the Implicit Association Test (IAT). For example, while Malinen and Johnston (26) find a negative implicit attitudes towards older workers using the age IAT they report no evidence of a negative explicit towards older workers. Similarly, Howell et al. (27) report a statistically significant divergence between explicit and implicit attitudes towards race when using the race IAT. In this literature the gap between implicit and explicit measures has been attributed to a social desirability bias which occurs when respondents give answers to questions that they believe will be viewed favourably by others, concealing their true opinions or experiences (28). As such, especially when considering socially sensitive subjects, implicit measures are better able to capture underlying attitudes, and be less prone of a social desirability bias, than explicit measures and are the preferred measure to explore these topics (29).

The most popular method for measuring implicit attitudes is the IAT (30). The IAT gives a measure of (positive or negative) implicit associations towards a particular group of people based on reaction times in assigning various stimuli (e.g., pictures of people with and without disabilities) together with positive or negative stimuli (i.e., good and bad words). The premise behind the IAT is that response times are slower when the stimuli combination is incongruent with the person's own implicit attitudes (i.e., people who see disability as a bad thing will take longer when putting disabled/good stimuli together than non-disabled/good). Research over the past 20 years has explored the distribution of implicit biases towards various characteristics including race, gender and age using the IAT (31). These variants of the IAT have been used to explore implicit bias in many different contexts with specialist populations, including police officers (32) and healthcare professionals (33, 34).

In the current paper we focus on the disability version of the IAT that provides our central framework for operationalising implicit bias towards people with disabilities [see (35), for an overview of the development of the disability IAT]. Wilson and Scior (29) provide a systematic review of studies that have measured implicit biases using the disability IAT. They report robust and consistent findings across the literature in terms of (often strong) anti-disability implicit attitudes. As such, we state our first hypothesis.

***Hypothesis 1*** *: Participants will implicitly prefer people without disabilities to those with.*

### Implicit bias in the workplace

Whilst attitudes towards race and gender have both been studied in workplace and employment settings, the disability IAT has not yet been examined in the business community. Malinen and Johnston (26) use a modified version of the age IAT with older worker/younger worker categories and find negative implicit attitudes towards older workers amongst (business) students. Further, Zaniboni et al. (36) demonstrate how both explicit and implicit stereotypes about older people lead to lower evaluations of fictional older candidates compared to younger ones. In a more field-based application, Rooth (37) presents evidence from a study in Sweden that suggests higher levels of negative implicit attitudes towards Arab-Muslim result in the lower probabilities of Arab-Muslims being offered a job. Similarly, Agerström and Rooth (38) demonstrate similar results showing that hiring managers with higher implicit attitudes against obese people were less likely to offer obese people interviews. Reuben et al. (39) use the gender-science IAT and find people with higher implicit associations between men and science (and women and arts) were more likely to choose a man to be their “employee” and perform a mathematics task on their behalf in a lab-based experiment.

Small- to Medium-sized Enterprises (SMEs) face considerably resource constraints compared to large enterprises. These resource constraints have a significant effect on SMEs ability to implement effective and robust Human Resource Management (HRM) processes (40).

Further, the issue of tackling bias amongst human resource managers has long been acknowledged within the academic literature [e.g., (41–43)]. We now state two further hypotheses.

***Hypothesis 2****: People working for large firms will have lower levels of implicit bias towards people with disabilities than those working for smaller firms*.

***Hypothesis 3****: People working in Human Resources or those making hiring decisions will have lower levels of implicit bias towards people with disabilities than those not working in HR or people not making hiring decisions*.

### Implicit disability bias and own health Status

One of the most consistent predictors of lower implicit attitudes across the literature is contact with people with disabilities. Indeed, Pruett and Chan (35) find the strongest predictor of disability IAT scores to be the “Contact with Disabled People Scale”. Further, Enea-Drapeau et al. (44) find that professional caregivers have lower bias towards people with Down syndrome in a modified version of the disability IAT. Harder et al. (45) present an analysis of over 300,000 responses to the disability IAT on the Project Implicit website and find the most consistent predictors of bias to be gender (women have lower bias) and having prior contact with people with disabilities. Aberson (46) also presents evidence from the disability IAT and sexuality IAT that having contact (with people with disabilities and gay people, respectively) results in lower negative implicit attitudes towards those, respectively. Further, Chowdhury et al. (47) present evidence that the use of a virtual reality disability simulator can reduce implicit bias towards people with disabilities.

It is well understood within the wider (i.e., non-disability) IAT literature that being “in-group” to a particular group reduces bias towards that group [see (27, 48) for examples with respect to race and gender]. However, we are unaware of any studies that look at the effect of own disability/health status on moderating implicit bias towards people with disabilities. Our study is the first to link a person's own health status (measured using the EQ-5D-5L) to their implicit bias towards people with disabilities. We now state our final hypothesis.

***Hypothesis 4:*** *People with worse overall health states will have lower levels of implicit bias towards people with disabilities than those with better overall health status*.

## Methods

### Data collection platforms

The survey and EQ-5D-5L data were collected using the LimeSurvey platform and the IAT data were collected using Millisecond's Inquisit platform. The IAT was a modified version of the off-the-shelf IATs available as part of the Inquisit library. It could be completed on either a computer, tablet or mobile phone using Windows OS, Mac OS or Android as appropriate. The entire procedure took an average of 16 min to complete.

Data collection was originally intended to be done in person using laptop computers at various business events across Cornwall and the two initial sessions of data collection reflect this (representing 9 of 108 responses). Subsequently, in light of COVID-19, the data collection process was converted into an entirely online platform. The study was granted ethical approval by [REDACTED FOR SUBMISSION] Research Ethics Committee and the approval was amended to reflect the change to online data collection ([REDACTED FOR SUBMISSION] Research Ethics Committee reference: e[REDACTED]002381). Participants first read through a general information sheet and then a consent statement that they agreed to before participating.

The information/instructions, the pre-questionnaire and the IAT procedures are provided in separate appendices (Supplementary File).

### Participants

The sample was drawn from the business community across Cornwall and the Southwest, UK, using snowball sampling. There are just over 25,000 businesses registered in Cornwall (49). The initial convenience sampling was done using local business networks (namely the Cornwall and Isles of Scilly Chamber of Commerce and Cornwall and Isles of Scilly Local Enterprise Partnership mailing lists which cover both small and large businesses across Cornwall), but there was also a snowballing effect at the organisational level as participants forwarded the study to their colleagues. Participants had to be over 18 years, and employees working in HR or those otherwise involved in hiring decisions were particularly encouraged to participate. Participants did not receive compensation for taking part. A power sample calculation based on using fixed effects ANOVA and with alpha significance criterion 0.05, standard power criterion of 80% and an effect size of 0.3 (representing moderate bias, see (50) gives a total required sample size of 90 participants using GPower (51).

### Procedures

The study was divided into 3 sections. Section 1 involves a survey with demographic and other contextual questions, in section 2 participants completed a Health Related Quality of Life (HRQoL) survey and finally in section 3 participants completed the IAT(s).

In section 1 of the study participants answered questions relating to demographics including whether the person considers themselves to have a disability and their experience of interactions with people with disabilities in the past (i.e., family members, colleagues, friends, etc.). We did not provide a definition of disability to participants to avoid any potential priming effects given the focus of the study is attitudes towards people with disabilities. Where applicable (for example, a question relating to explicit attitudes), these questions were modelled on the questions used on the Project Implicit portal, though additional contextual questions relating especially to the workplace were also included. Thus, the survey also asked people whether they are involved in recruitment or retention decisions, and if so how long they have been involved. Additional questions asked about their firm's sector of the economy and number of employees.

In section 2 of the study participants completed a Health Related Quality of Life questionnaire (EQ-5D-5L) that is used widely within Health Economic evaluations. The EQ-5D-5L (52, 53) is a measure of Quality of Life across 5 dimensions (mobility, self-care, usual activities, pain/discomfort and anxiety/depression) each with 5 possible levels (no problems, slight, moderate, severe and extreme problems). We apply the National Institute of Health and Care Excellent (NICE) guidance (54) and calculate the EQ-5D-5L index values from these responses using a preference-based tariff derived from members of the general public and the Crosswalk Index Value Calculator using the United Kingdom value set (55). It should be noted that the EQ-5D may not be able to properly capture the health status of people with disabilities (56). Further, the EQ-5D is situated within the medical model of disability—focusing on physical impairments. Nonetheless, it is a well cited general measure of HRQoL (57).

**Figure 1:**
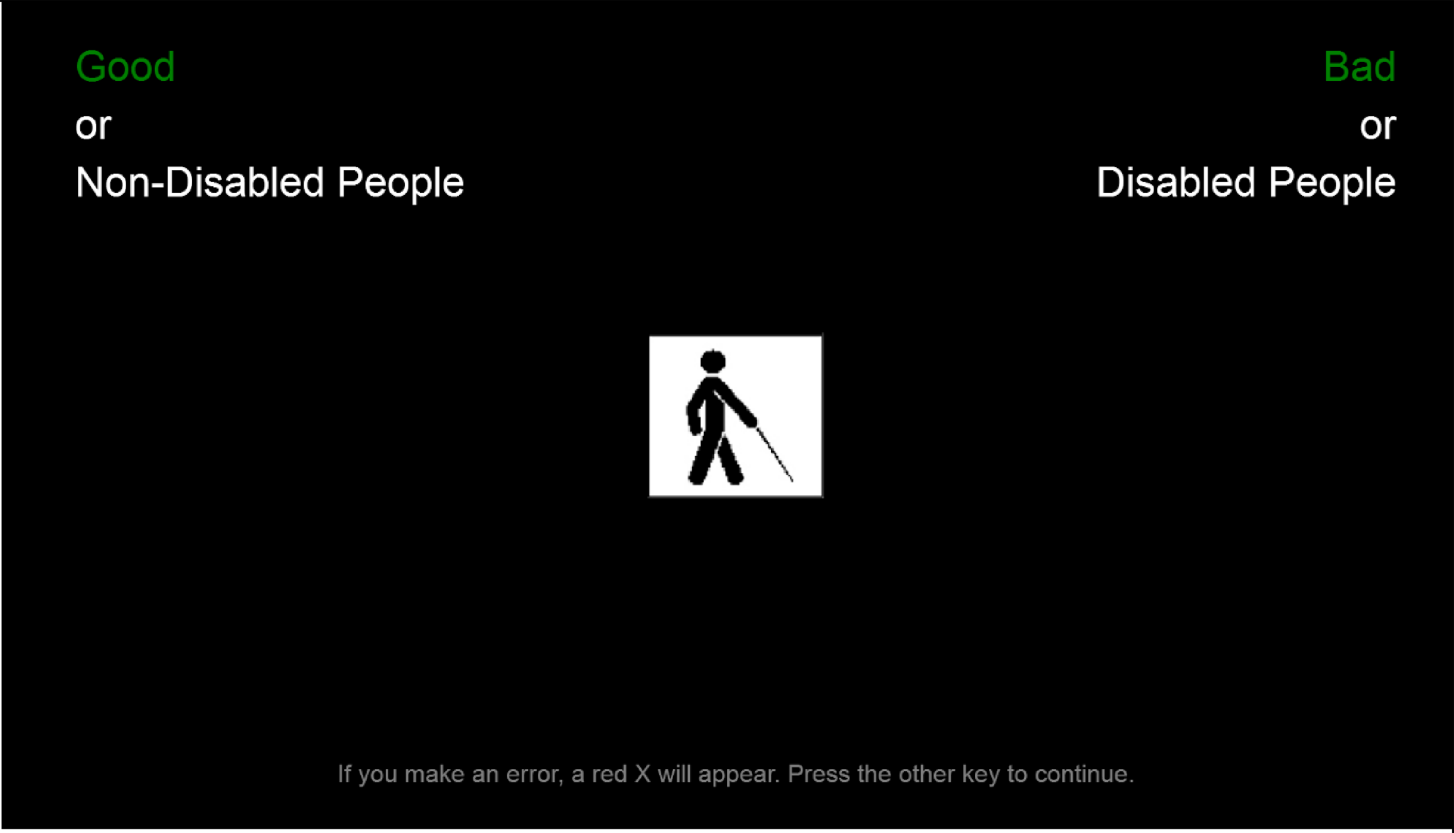
Screenshot of the disability IAT. Note: Since the preferred terminology in the UK is “disabled people” rather than “people with disabilities”, the term “disabled people” was used in the materials presented to participants.

In section 3 participants completed an online version of the disability IAT. Again, no definition or explanation of disability is given to avoid potential priming effects. Prior to completing the IAT, participants decided whether or not they would see their results at the end of the IAT. This option was provided to counter the potential for negative or defensive reactions to the results that have been reported by others (58). As mentioned above, the IAT involves participants correctly assigning various stimuli to pre-determined categories. The stimuli were either images representing disabled/non-disabled categories or words representing good/bad categories (see Figure 1). While the good/bad categories remained fixed, the disabled/non-disabled categories swapped over halfway through. The full set of stimuli used can be viewed in Table 1—as can be seen, the stimuli all related to physical, visible disabilities. Whether people take part in good-disabled or bad-disabled trials first is randomised to prevent order effects. The measure of implicit attitudes is taken from the difference in reaction times.

**Table 1:** Stimuli used for the disability IAT.

Category	Items
Good	Adore, Pleasure, Lovely, Delightful, Glad, Friendship, Attractive, Excellent
Bad	Dirty, Abuse, Annoy, Scorn, Gross, Hatred, Awful, Detest
Non-disabled people	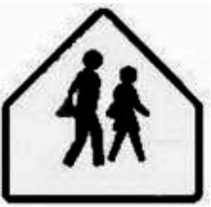 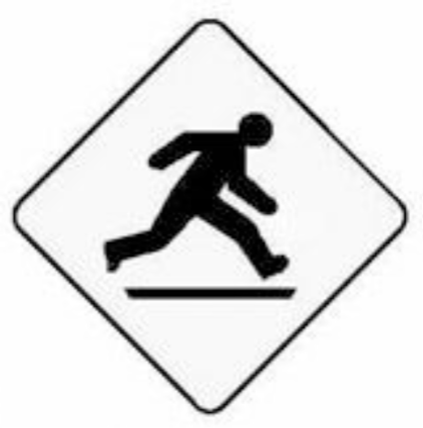 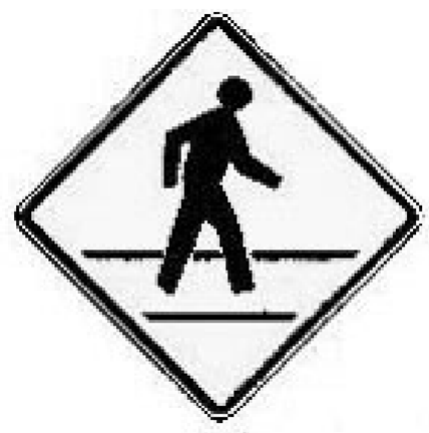 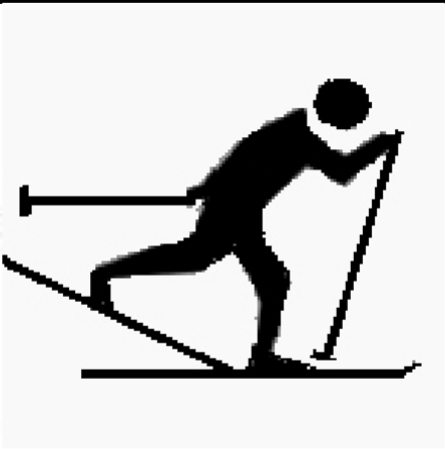
Disabled people	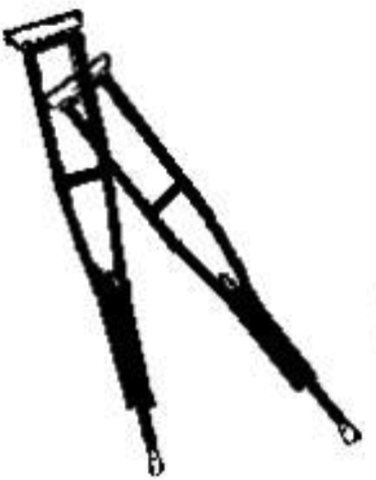 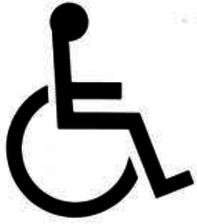 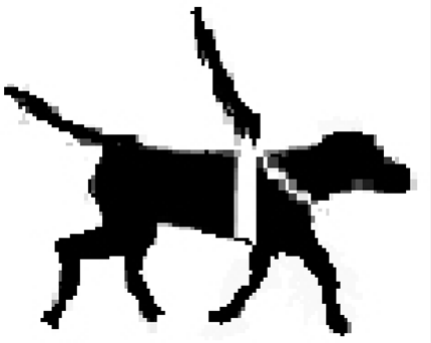 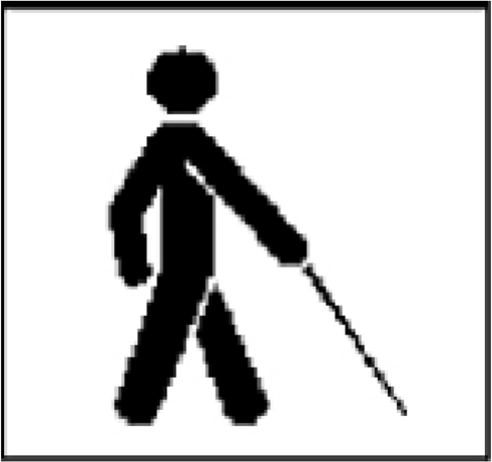

**Note:** These are the terminologies and stimuli as presented to participants.

### Calculation of the IAT D-scores

Participants reaction times to the stimulus are standardised using the improved scoring algorithm due to Greenwald et al. (59) and the resulting “D-score” ranges between −2 and 2. Positive scores represent anti-disabled bias and negative scores represent pro-disabled bias. Anything above 0.15 (below −0.15) is taken to be a slight bias, above 0.35 (below −0.35) is a moderate bias and above 0.65 (below −0.65) is a strong bias. Less than 0.15 is no bias.

### Validity and reliability of the IAT D-scores

There is a substantial body of literature investigating the validity and reliability of the IAT. In one of the first such studies, Cunningham et al. (60) present evidence that the IAT correlates with other measures of implicit attitudes. Further, Hoffman et al. (61) and Greenwald et al. (62) conduct meta-analyses to systematically combine the results of previous research to assess the ability of the IAT to predict behaviour. Their results suggest that the IAT has good predictive validity and predicts behaviour better than explicit attitude measures. A subsequent meta-analysis by Kurdi et al. (63) investigates the validity of the IAT to predict explicit measures and similarly finds the IAT has good predictive validity. Pruett and Chan (35) look specifically at the disability IAT and confirm its ability to distinguish between positive and negative associations towards people with disabilities. Nonetheless, the validity of the IAT remains a much debated topic within the literature [see (64, 65)].

There is also evidence that the IAT is robust to being falsified. Banse et al. (66) present evidence that participants are unable to falsify a positive implicit association with respect to homosexuality when instructed to. However, whilst supporting the results of Banse et al. (66), Steffens (67) presents evidence that participants may be able to fake positive implicit associations when given repeated exposure to the IAT procedure.

### Statistical methods

Hypothesis 1 is tested using the using one-way ANOVA using the IAT D-scores. Hypotheses 2–4 are tested directly using ANOVA analysis to compare IAT results between different subgroups. Linear regression was used to test hypothesis 4. Additionally, we undertook exploratory regression analysis to explore other factors that might impact on implicit bias.

## Results

### Descriptive statistics

Table 2 provides an overview of the descriptive statistics for our sample. In total, 108 participants took part in the study. The average age of participants was 44 years and 70% of participants were female. Half (50%) of the sample was based in Cornwall, UK. Further, 16% of participants self-reported having a disability. Finally, 44% self-reported being involved in making recruitment and/or retention decisions in their current role and 50% self-reported working for a small-to-medium sized enterprise (SME; less than 250 employees).

**Table 2:** Participant descriptive statistics (*N* = 108).

Variable	Value (in years or as percentage)
Age	44 years (SD: 11.93, range: 18–78)
Gender (1 = Female, 0 = Male)	76/108 (70%) (SD: 0.46)
County (1 = Cornwall, 0 = Other)	54/108 (50%) (SD: 0.50)
Disability (1 = Yes, 0 = No)	17/108 (16%) (SD: 0.37)
Involved in hiring decisions (1 = Yes, 0 = No)	48/108 (44%) (SD: 0.50)
Works for large employer, 250 + employees (1 = Yes, 0 = No)	54/108 (50%) (SD: 0.50)

Standard Deviations (SD) of the mean values are given in brackets and the range is also given for the age variable.

### Disability IAT

*Do participants implicitly prefer people without a disability to people with a disability?*

**Figure 2:**
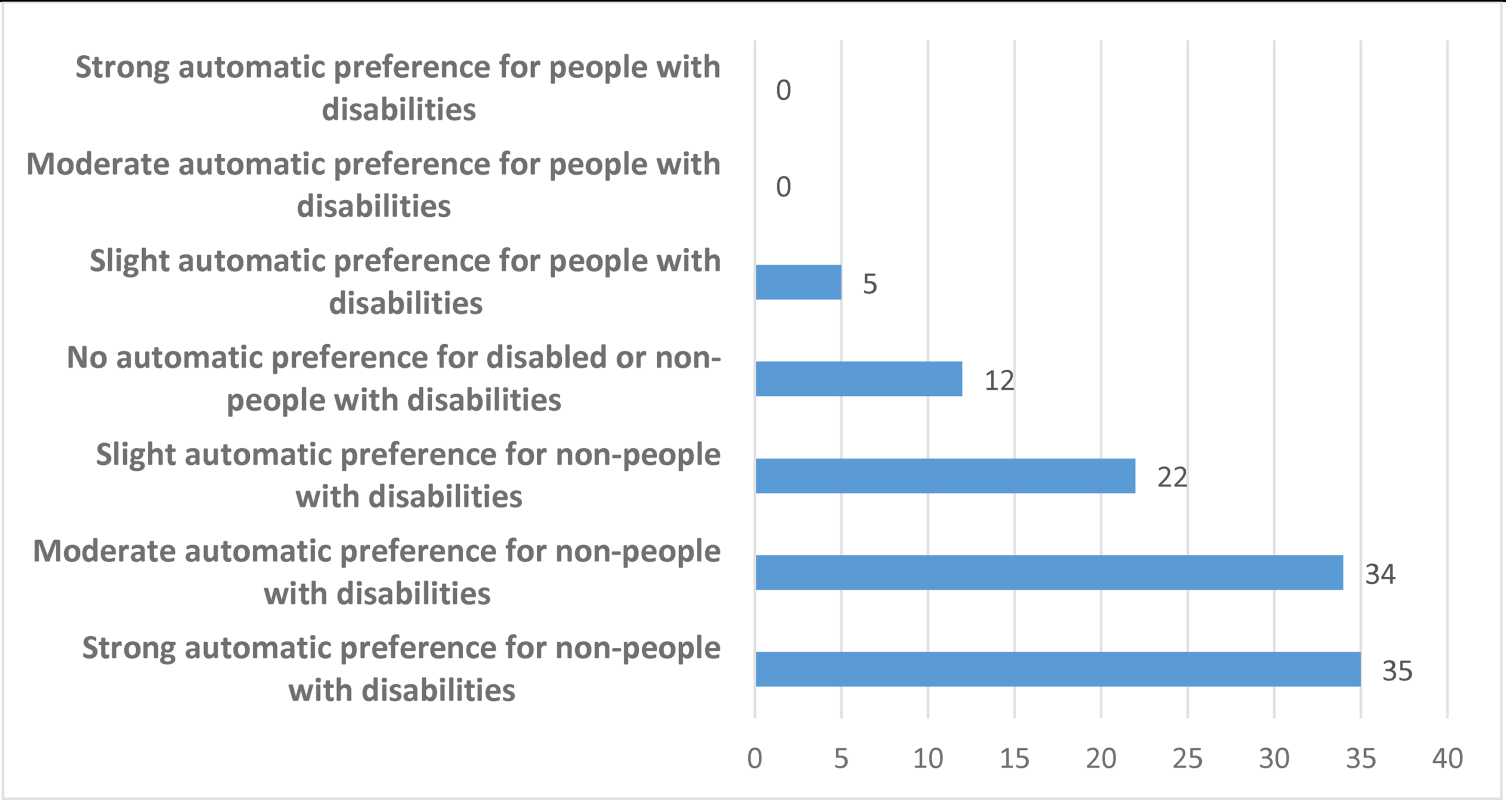
Distribution of disability IAT scores (*N* = 108).

Figure 2 shows the distribution of D-scores according to the usual classification described above, giving no bias, slight bias, moderate bias and strong bias categories in both directions. 74% (80/108) of participants show either a slight, moderate or strong bias against people with a disability in favour of those without. Only 7% (8/108) of participants showed a bias in favour of people with disabilities and 19% (20/108) showed no bias.

*Result 1: The majority of participants show implicit preferences for people without disabilities compared to people with disabilities*.

*Do workers in large firms have lower levels of implicit bias towards people with disabilities?*

**Figure 3:**
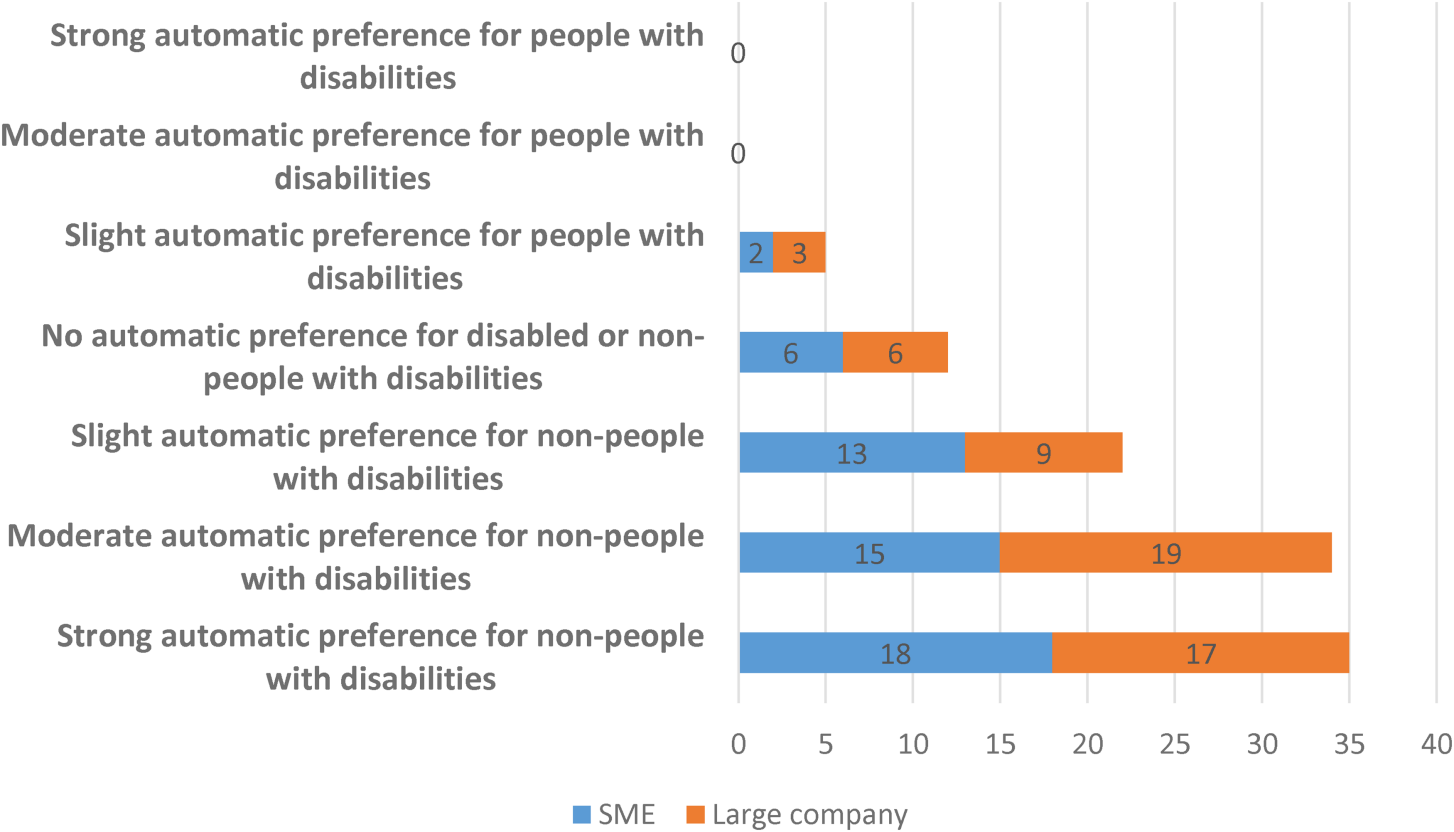
Distribution of IAT scores categorised by whether someone works for an SME or a large company (*N* = 108).

We performed a one-way ANOVA on the IAT D-scores to examine the effect of being involved in making recruitment and retention decisions. We also looked at whether there is any effect of working for a large company (see Figure 3). We find that there is no significant difference depending on whether a participant works for a large company or not (Table 3; *F* = 0.08, *p *= 0.778).

**Table 3:** Effect of company size on disability IAT scores (*N* = 108).

Works for SME	D-score Mean	S.D	*N*	ANOVA
No	0.527	0.384	54	*F* = 0.08, *p *= 0.778
Yes	0.506	0.382	54	

*Result 2: There are no significant differences in implicit attitudes towards people with disabilities between people who work for large companies compared to SMEs*.

*Do people working in Human Resources or making hiring decisions have lower levels of implicit bias towards people with disabilities?*

**Figure 4:**
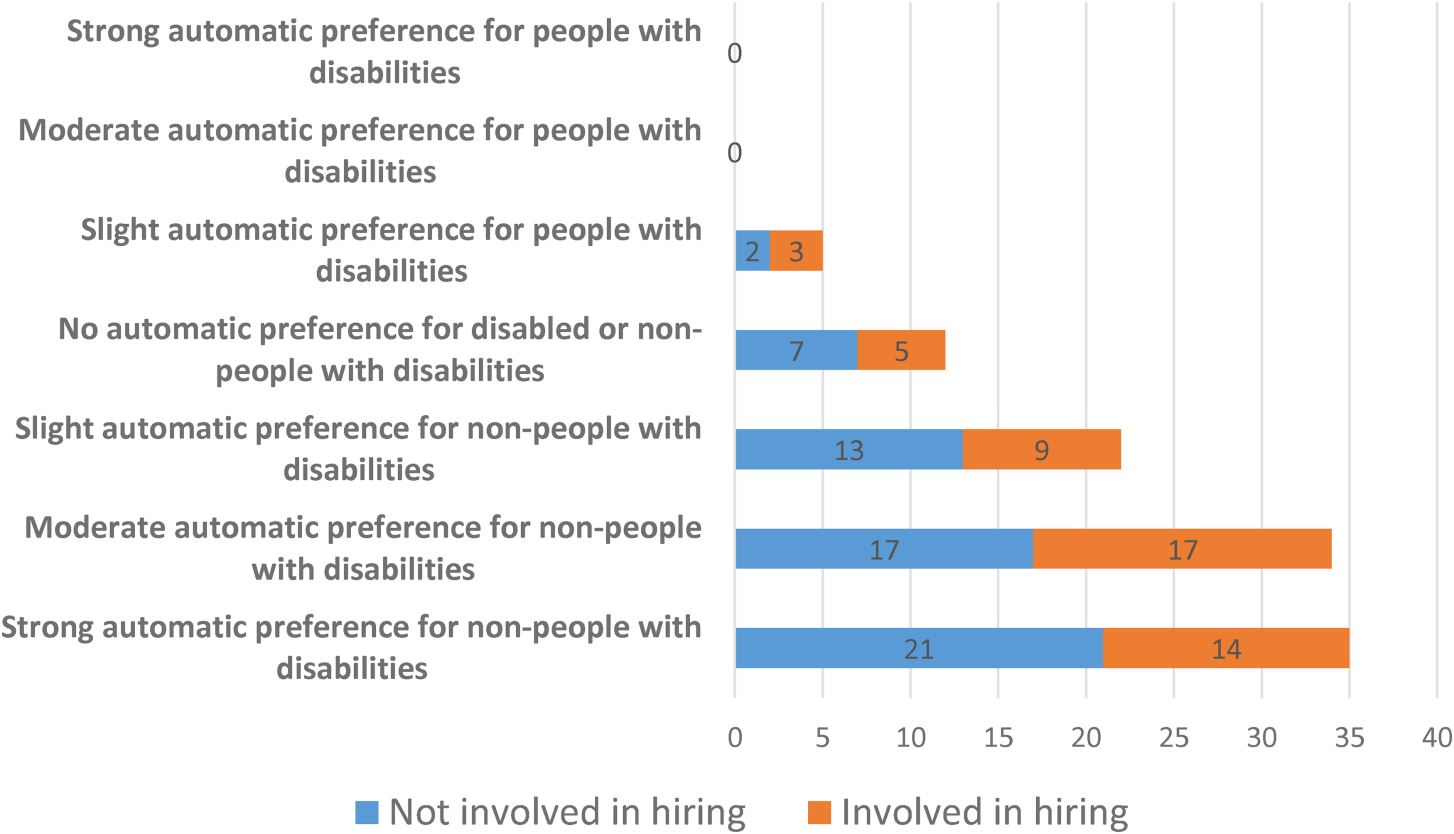
Distribution of disability IAT scores categorised by whether someone is involved in making hiring decisions or not (*N* = 108).

The ANOVA results suggest that being involved in making recruitment and/or retention decisions does not have a significant effect (Table 4: *F* = 0.02, *p* = 0.893). This can also be seen in Figure 4. It should also be noted that these results are robust if we only look at people who have been involved in recruitment and/or retention decisions for at least 2 years (*F* = 0.02, *p* = 0.878).

**Table 4:** Effect of being involved in recruitment and/or retention decisions on disability IAT score (*N* = 108).

Involved in hiring decisions	D-score Mean	S.D	*N*	ANOVA
No	0.521	0.367	60	*F* = 0.02, *p *= 0.893
Yes	0.511	0.403	48	

For both hiring decisions and size of the firm, the results are robust to running a multivariate ANOVA (MANOVA, available in Supplementary Appendix S2). Similarly, the results are robust if we look at people involved in recruitment and/or retentions decisions that also work for a large company (23 of our 108 participants)—this is also not significant (*F* = 0.15, *p *= 0.703).

*Result 3: There are no significant differences in implicit attitudes towards people with disabilities between people involved in making hiring decisions and people who are not*.

*Do people with worse overall health states and women have lower levels of implicit bias towards people with disabilities?*

In order to further explore the factors that affect IAT scores, we conduct regression analysis that can be seen in Table 5. We do find a significant effect for both a self-reported measure of a disability and the EQ-5D-5L Crosswalk Index Value, both of which lead to significantly lower bias against people with disabilities. We find that women have significantly lower bias against people with disabilities than men. We do not find an effect of having prior contact with people with disabilities.

**Table 5:** Regression analysis with disability IAT score as dependent variable.

**Dep. Var.:** D-score (*N* = 108)	Coefficient	*p*-value
Age *(Age in years)*	−0.004 (0.004)	0.283
Gender *(1 = female, 0 = male)*	−0.201 (0.090)	**0**.028**
Disability *(1 = yes, 0 = no)*	−0.259 (0.123)	**0**.038*
EQ-5D-5L *(UK Crosswalk Index Value)*	−0.517 (0.222)	**0**.022**
Disability contact *(0 = none, 7 = most)*	0.012 (0.029)	0.676
Explicit attitudes *(1 = strong pro-disabled, 7 = strong anti-disabled)*	0.031 (0.052)	0.554
Involved in hiring decisions *(1 = yes, 0 = no)*	−0.021 (0.135)	0.851
Involved in hiring decisions at least 2 years *(1 = yes, 0 = no)*	−0.077 (0.125)	0.541
Works for large company *(1 = yes, 0 = no)*	−0.077 (0.125)	0.584
Involved in hiring decisions AND works for large company *(1 = yes, 0 = no)*	0.121 (0.159)	0.447
Cornwall *(1 = yes, 0 = no)*	0.109 (0.077)	0.159
Constant	1.143 (0.349)	**0**.001**

*N* = 108, **p < 0.05, *p < 0.1, OLS estimates, robust standard errors presented in parentheses below the coefficients. Disability contact refers to the number of possible categories chosen amongst friends, parent, child, sibling, colleague, spouse, other.

*Result 4: People with worse overall health states and women have significantly lower levels of implicit bias towards people with disabilities compared to people with better overall health states and men*.

The regression also confirms that neither working for a large company nor being involved in hiring decisions has any effect on implicit bias against people with disabilities. We similarly find no effect for age, explicit attitudes or being in Cornwall. Therefore, in our sample, the main factors that affect implicit bias against people with disabilities are gender and a participant's own disability/health status.

## Discussion

### Implicit bias in the workplace

We find substantial levels of implicit bias against people with disabilities that are very much in line with the existing literature and publicly available data using the disability IAT with the general public. We find no significant differences in the levels of implicit bias for the disability IAT depending on whether the person is involved in recruitment and/or retention decisions. Additionally, we provide the first examination of the effect of firm size (SME or not) on IAT results and find no significant differences. We also find that women have significantly lower biases against people with disabilities than men.

In terms of the specific business factors, large companies spend significant amounts of money on EDI and unconscious bias training (UBT)—up to $8 billion each year in the USA (68). Similarly, there has been a substantial increase in the number of diversity and inclusion-based job roles over recent years (a global rise of 71% from 2015 to 2020; see (69)). People involved in recruitment and retention decisions—especially HR professionals—are also often specifically trained in EDI issues given their remit of ensuring compliance with relevant legislation [in our context, the Equality Act 2010 (UK)]. We therefore find it surprising that neither working for a large company nor being involved in HR have a significant effect on implicit attitudes towards people with disabilities, which require deeper and more structural reimagining of paradigms and modes of thinking with respect to disability to meaningfully change. This may suggest that the current efforts of large companies compared to SMEs are not effective at reducing implicit bias and that current strategies require rethinking.

### Representation of people with disabilities

We find that a participant's own disability/health status is associated with lower implicit biases, as anticipated from other IAT literature on in-group effects. To our knowledge this is the first IAT study that has collected participant's health status using validated methods from within the Health Economics literature (i.e., the EQ-5D-5L). This finding is consistent with the finding for other IATs (e.g., race and gender) which show that people exhibit lower bias towards groups they belong to (i.e., in-group). We recommend that future work should aim to explore the relationship between a participant's own health status and implicit bias against people with disabilities in more depth. As such, our results suggest greater disability representation within the workplace should lower average levels of implicit bias towards people with disabilities.

In our study, of the 48 people who reported being involved in hiring decisions and/or HR, only 6% (3 people) reported having a disability. On the other hand, of the 60 not involved in hiring decisions and/or HR, 23% (14 people) reported having a disability—which is broadly in line with the national average. This difference is statistically significant and suggests that people with disabilities are underrepresented in the HR profession and in making hiring decisions.

Though our study does not find that implicit biases are lower for participants with prior contact with people with disabilities there is strong evidence for such a link in the literature (29, 46), further indicative of the positive benefits of more disability representation in the workplace. As such, it is clear that there is a need for greater disability representation—especially within HR and at senior levels where people make hiring decisions—and that bridging this gap in representation may improve both attitudes towards people with disabilities and the disability employment gap. Addressing negative attitudes towards people with disabilities in the workplace should be a high priority for policy makers interested in the disability employment gap.

### The disability IAT

It is worth noting that all the disabled stimuli used within the disability IAT are related to physical and visible disabilities. It does not therefore capture implicit biases towards invisible disabilities, including mental health and learning disabilities. Further, all the stimuli involve people using some form of equipment, i.e., a wheelchair or white cane, when in reality most people with a disability don't require any specialist equipment. As such, the disability IAT itself may reinforce particular notions and stereotypes about what a person with a disability is “supposed” to look like. The stimuli are particularly medicalised representations of people with disabilities and, having been in use since the development of the disability IAT in the early 2000s, may need to be revisited. For example, the recent development of more focused IATs has tended towards the use of word based stimuli only, e.g., the autism IAT [see (70–72)].

### Limitations

Both of our measures—the EQ-5D and the IAT—are the subject of debate within the disability literature with respects to their validity in representing the lived experiences of people with disabilities. Further research towards the representation of disabled people within the development of these measures is encouraged to improve the relevance of these measures in terms of discourse around disability.

Our recruitment strategy was effective in targeting people who are involved in hiring decisions and/or who work for SMEs. The descriptive statistics for this subsample are reported in an Supplementary Appendix S1 and are almost identical to the full sample. Interestingly, of the 108 participants who completed the disability IAT, 106 chose to see their results at the end and only 2 chose not to. It is unsurprising that the majority of respondents are female, since this is in keeping with the fact that HR remains a female dominated industry (the Chartered Institute of Personnel and Development (CIPD) in the UK has 63% female membership (73).

We acknowledge, however, that our approach to recruit respondents to the IAT may have resulted in self-selection and attracted people with a particular interest in disability inclusion or inclusion more generally. This may lead to an underestimation of the true levels of unconscious bias towards people with disabilities within the business community.

Further, we lack data about the extent to which participants have previously taken part in EDI initiatives or unconscious bias training (UBT). Similarly, there may be other meaningful differences—beyond propensity to undertake EDI training or UBT—between large companies and SMEs or between people who work in HR/make hiring decisions and those who do not (although there are no substantial differences in demographics within our sample). Nonetheless, this only affects the interpretation laid out in the discussion above and not the substance of the results.

Whilst our study provides some initial findings around the disability IAT and the workplace by providing a sample of the business community, future research may wish to replicate studies with other versions of the IAT correlating the IAT scores with hiring decisions either in the real world or in a lab-based setting.

## Conclusion

We use the disability IAT to study attitudes towards people with disabilities within a sample of the business community from across the South West of the UK. We find significant negative attitudes towards people with disabilities, in line with previous studies with general populations.

Our results highlight the need for greater disability representation within the workplace and a critical rethink of current approaches to address negative attitudes towards people with disabilities—both within the workplace and beyond.

## Data Availability

The datasets presented in this study can be found in online repositories. The names of the repository/repositories and accession number(s) can be found below: https://reshare.ukdataservice.ac.uk/855417/.
