# Supplementary material for: The importance of disability representation to address implicit bias in the workplace
Source: Front Rehabil Sci. 2023 Mar 22;4:1048432. doi: 10.3389/fresc.2023.1048432 (PMC10073421; doi:10.3389/fresc.2023.1048432)

**APPENDICES**

**APPENDIX 1: DESCRIPTIVE STATISTICS FOR AGE IAT**

| **Table A: Participant descriptive statistics for age IAT (N=50)** | |
| --- | --- |
| **Variable** | **Value** |
| Age | 44 years  (SD: 12.91, range: 18-78) |
| Gender (1=Female, 0=Male) | 68%  (SD: 0.47) |
| County (1=Cornwall, 0=Other) | 44%  (SD: 0.50) |
| Disabled (1=Yes, 0=No) | 14%  (SD: 0.35) |
| Involved in hiring decisions (1=Yes, 0=No) | 46%  (SD: 0.50) |
| Works for large employer, 250+ employees (1=Yes, 0=No) | 54%  (SD: 0.50) |

**APPENDIX 2: ADDITIONAL MANOVA ANALYSIS**

| **Table B: MANOVA analysis on firm size and being involved in hiring decisions (N=108)** | | | | |
| --- | --- | --- | --- | --- |
| **Source** | **Statistic** | | **F** | ***p*** |
| Model | W | 0.999 | 0.05 | 0.951 |
|  | P | 0.001 | 0.05 | 0.951 |
|  | L | 0.001 | 0.05 | 0.951 |
|  | R | 0.001 | 0.05 | 0.951 |
| Large company | W | 0.999 | 0.08 | 0.775 |
|  | P | 0.001 | 0.08 | 0.775 |
|  | L | 0.001 | 0.08 | 0.775 |
|  | R | 0.001 | 0.08 | 0.775 |
| Involved in hiring decisions | W | 0.999 | 0.02 | 0.885 |
|  | P | 0.001 | 0.02 | 0.885 |
|  | L | 0.001 | 0.02 | 0.885 |
|  | R | 0.001 | 0.02 | 0.885 |
| W = Wilks’ lambda, P = Pillai’s trace, L = Lawley-Hotelling trace, R = Roy’s largest root  F = F-statistic, *p=*=p-value | | | | |

**APPENDIX 3: AGE IAT STIMULI**

| **Table C: Stimuli used for the age IAT** | |
| --- | --- |
| **Category** | **Items** |
| **Good** | Joyous, Joyful, Happy, Attractive, Laughing, Beautiful, Lovely, Cherish |
| **Bad** | Tragic, Sickening, Scorn, Failure, Negative, Hate, Horrific, Awful |
| **Young** | 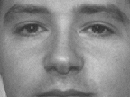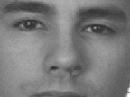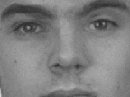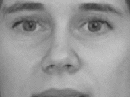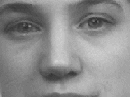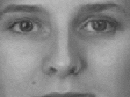 |
| **Old** | 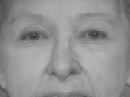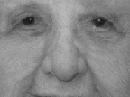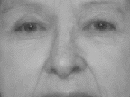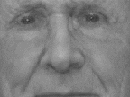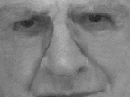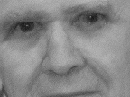 |

**APPENDIX 4: INFORMATION AND CONSENT STATEMENT**

The Inclusivity Project Research Study - Information for Participants

**What is the aim of the project?**

It has been reported that many businesses across the United Kingdom are struggling to recruit the staff they need.  At the same time older workers and those workers with disabilities or long-term conditions are underrepresented in the work force.  The Inclusivity Project has been developed to explore the barriers and issues faced by small- to medium-sized enterprises (SME) in recruiting and retaining older workers and those with disabilities or long-term conditions.

**What will participants be asked to do?**

You will be asked to take part in an online survey exploring your beliefs and attitudes towards age and disability as well as some standard questions about yourself. As part of the survey you will be asked to sort a set of pictures and words as fast as you can to explore your views. There are no right or wrong answers to these questions. In order to participate in the sorting exercise you will be required to install the Inquisit Player plugin, full instructions are given on how to do this at the appropriate point.

**Time commitment** 

The survey will take approximately 20-30 minutes to complete.

**Compensation**

There is no payment for the participation in the survey as we hope the survey will give you a chance to reflect on issues surrounding the recruitment and retention of disabled and/or older workers.  

**Please note that by clicking continue and completing the survey you agree to the below. Please read the following carefully before continuing.** 

I understand that my participation is voluntary and that I am free to stop the survey at any point before completing the survey.

I understand that anonymised data will be used by the research team from the **[REDACTED FOR SUBMISSION]**. This data will be used to produce academic publications and reports for businesses and policy makers.

I understand that the data collected during the studied will be retained in secure storage and that my identity will not be linked to any publications or reports resulting from the survey. The data will be stored for 5 years after which it will be destroyed.

**If you have any questions or feedback, please contact [REDACTED FOR SUBMISSION] at [REDACTED FOR SUBMISSION]. This project has been granted ethical approval by the [REDACTED FOR SUBMISSION] reference [REDACTED FOR SUBMISSION].**

**Instructions**

You will first be asked to fill in a short questionnaire that asks you about;

- Some basic demographic information
- The sector you work in and your job role responsibilities
- Your current health status

You will then be asked to complete a short exercise designed to examine any implicit associations you may have towards older people and people with disabilities.

You will be given additional instructions on this exercise after the questionnaire.

Research shows that these implicit associations can represent a significant and real barrier to the employment of older people and people with disabilities.

As such, the results of this study will then be used to inform the design of interventions and policies to minimise the effect that such implicit associations can have in the workplace with a view to creating more inclusive workplaces.

Finally, a reminder that all your results will be anonymised at the time of collection and you therefore will not be identifiable in any way.

**APPENDIX 5: PRE-QUESTIONNAIRE**

What is your gender?

Which county do you live in?

Do you consider yourself to have a disability?

What kind of experience do you have interacting with people with disabilities? (You may select more than one answer).

What kind of experience do you have of interacting with older people? (You may select more than one answer).

Which sector do you work in? (Please choose the most applicable category).

How many people work for your employer, across all of their sites and locations, and including yourself?

Are you involved in recruitment and/or retention decisions in your current role?

Which statement best describes you?

Which statement best describes you?

**APPENDIX 6: IAT PROCEDURES**

Participants are instructed to assign the stimuli to the correct categorises as quickly as possible. The IAT consists of 7 blocks, the 1^st^, 2^nd^ and 5^th^ of which are practice blocks. Half of participants saw good-non-disabled/bad-disabled (known as the ‘congruent block’) first and the other half saw good-disabled/bad-non-disabled first (known as the ‘incongruent block’) with random assignment. In blocks 6 and 7, the order is reversed. An overview of the 7 blocks can be found in Table Y. Participants also decided at the start whether they wanted to see their results at the end or not.

| **Table Y: Overview of the disability IAT** | | | | |
| --- | --- | --- | --- | --- |
|  | **Order 1 (Congruent first)** | | **Order 2 (Incongruent first)** | |
| **Block 1 – 20 trials** | Non-Disabled | Disabled | Disabled | Non-Disabled |
| **Block 2 - 20 trials** | Good | Bad | Good | Bad |
| **Block 3 - 20 trials** | Good | Bad | Good | Bad |
| **Block 4 - 40 trials** | Non-Disabled | Disabled | Disabled | Non-Disabled |
| **Block 5 - 20 trials** | Disabled | Non-Disabled | Non-Disabled | Disabled |
| **Block 6 - 20 trials** | Good | Bad | Good | Bad |
| **Block 7 - 40 trials** | Disabled | Non-Disabled | Non-Disabled | Disabled |

[This appendix provides indicative screenshots of the IAT used in the study, including the additional first page asking whether participants want to see their results. A full set of screenshots and/or the Inquisit files are available on request.]


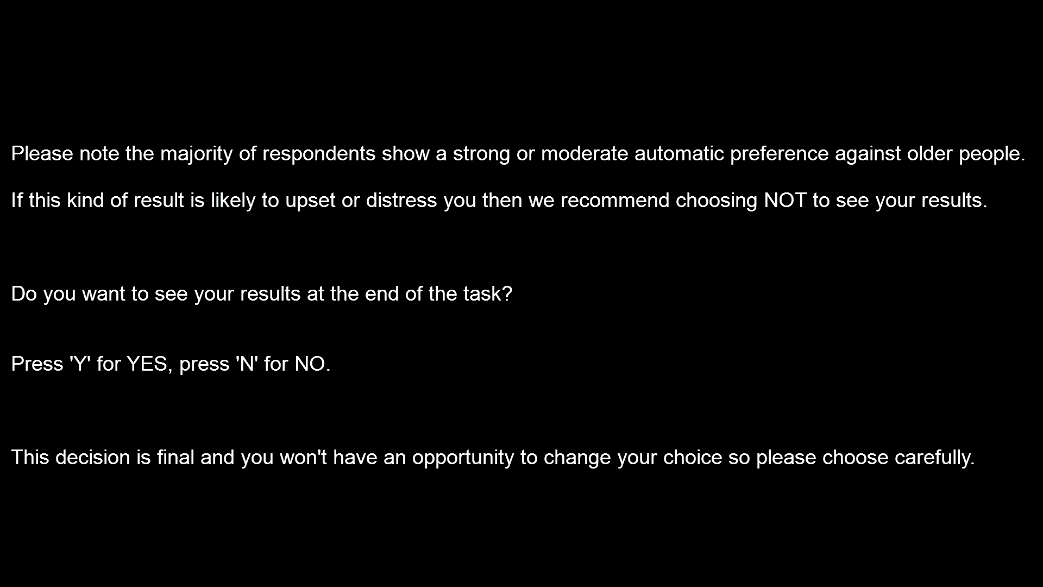


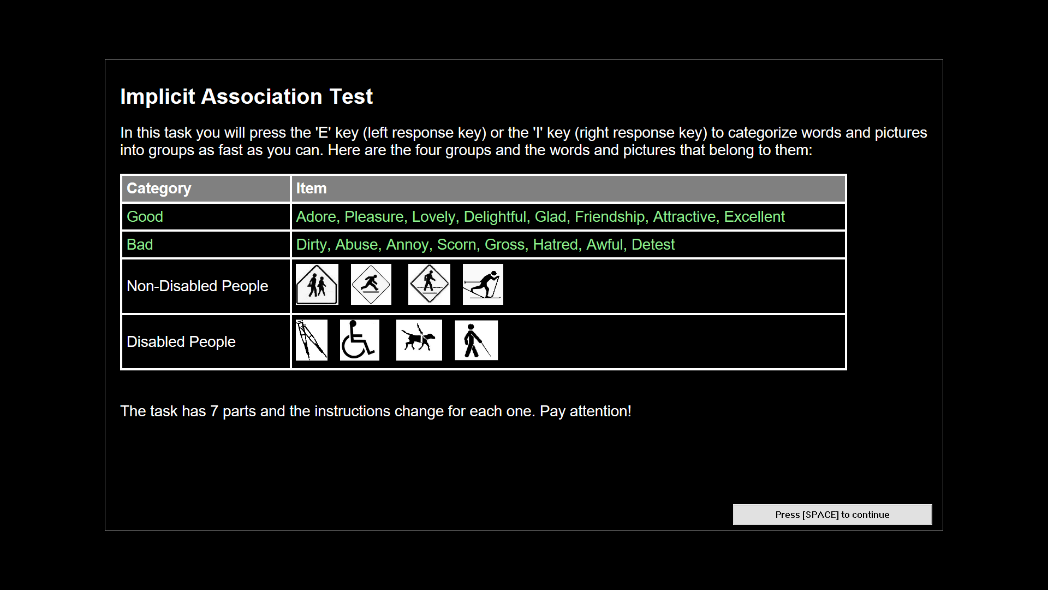

Supplement: Supplementary file 1 [file Datasheet1.docx]
